# Boosting Microwave Absorption via Hollow CoFe/CoFe_2_O_4_ Nanoparticles Anchored on Lotus-Leaf-Derived Biochar

**DOI:** 10.3390/nano16201323

**Published:** 2026-10-20

**Authors:** Feng Han, Maomao He, Hongchun Yu, Luran Zhang

**Affiliations:** National Center for International Research on Photoelectric and Energy Materials, School of Materials and Energy, Yunnan University, Kunming 650500, China; feng0718228@yahoo.co.jp (F.H.); hmm0513@163.com (M.H.); 5198798462@163.com (H.Y.)

**Keywords:** biomass charcoal, hollow structure, electromagnetic wave absorption, multi-interface, CoFe/CoFe_2_O_4_/C

## Abstract

Biochar composites are promising for electromagnetic wave absorption due to their renewability and low cost, but performance optimization remains a challenge. Here, hollow CoFe/CoFe_2_O_4_ composite nanoparticles, in which the CoFe and CoFe_2_O_4_ phases coexist, are grown in situ on lotus-leaf-derived porous carbon via a Kirkendall-type process during heat treatment. The optimized composite (pyrolyzed at 800 °C) exhibits a saturation magnetization of 69.20 emu/g and a coercivity of 482 Oe; a calculated minimum reflection loss (*RL*_min_) of −71.4 dB is obtained at a matching thickness of 2.70 mm, with an effective absorption bandwidth (EAB) of 5.68 GHz at 1.70 mm, covering most of the Ku band. The enhanced microwave absorption performance is attributed to the synergy of dielectric and magnetic losses, polarization relaxations, and improved impedance matching, together with multiple scattering within the hollow structure. In addition, the exchange coupling between the FeCo and CoFe_2_O_4_ may contribute to magnetic dissipation, while hollow cavities may promote internal reflections for further energy attenuation. This work provides a biomass-derived route to high-performance biochar-based absorbers competitive with synthetic counterparts.

## 1. Introduction

The widespread use of electronic devices has raised growing concerns about electromagnetic radiation and its effects on human health and equipment performance [1,2,3], driving the demand for high-performance electromagnetic wave (EMW) absorbers with lightweight, thin, broadband, and strong-attenuation characteristics [4,5,6].

Biochar, a carbon material derived from renewable biomass (e.g., cellulose and lignin) via pyrolysis, has attracted interest in EMW absorption due to its low density, good conductivity, and naturally porous structure [7,8,9]. Lotus leaf, in particular, stands out as a biomass precursor: its leaf tissue architecture is largely preserved after pyrolysis, giving a naturally porous carbon scaffold that favors the uniform dispersion of nanoparticles and multiple scattering of incident EMW. However, the high permittivity of biochar often leads to impedance mismatch with free space, limiting attenuation efficiency. To address this issue, magnetic components are incorporated to form heterostructures that combine dielectric and magnetic losses, thereby optimizing impedance matching and enhancing energy dissipation [10,11]. For instance, Fan et al. prepared Fe@Fe_3_C/C composites from alfalfa, achieving 5 GHz bandwidth and −39.67 dB at 1.50 mm [12]; Zhang et al. fabricated Co/C scaffolds from phragmites, reaching 4.29 GHz bandwidth and −52.42 dB at 1.44 mm [13]. Despite notable progress through compositional engineering, balancing broadband absorption with high loss efficiency remains a challenge.

In addition to these single-metal systems, bimetallic alloy/carbon composites—such as CoFe/porous carbon [14], NiCo_2_/rice husk porous carbon [15], carbon nanofibers embedded with Fe–Co alloy nanoparticles [16] and hollow CoFe_2_O_4_/CoFe@C microspheres [17]—have been reported to deliver strong and broadband absorption, further demonstrating the potential of bimetallic architectures.

The FeCo bimetallic system offers a promising platform, combining excellent magnetic permeability, saturation magnetization, and electrical conductivity—all favorable for EMW attenuation [18,19,20]. The solvothermal route was selected because of its mild reaction conditions, good compositional controllability, and scalability; moreover, the oxygen-containing groups on the biochar surface can chelate the metal precursors, favoring stable in situ anchoring of the nanoparticles on the carbon scaffold. Additionally, the abundant oxygen-containing groups on biochar surfaces can chelate metal ions, facilitating stable anchoring of nanoparticles within the carbon framework. Beyond composition, architectural design is equally important: hollow structures not only reduce density but also introduce multiple scattering and reflection pathways, effectively broadening absorption bandwidth without sacrificing lightweight characteristics [21,22].

In this work, porous biochar was prepared from natural lotus leaves via KOH activation and gradient pyrolysis. Hollow CoFe/CoFe_2_O_4_ particles were then grown in situ on the carbon scaffold through a solvothermal–pyrolysis synergistic strategy. This magnetic composite combines the high saturation magnetization of the FeCo alloy with the high magnetocrystalline anisotropy of the CoFe_2_O_4_ ferrite; The interfacial exchange coupling between the two magnetic phases may contribute to the magnetic loss. The hollow architecture may be related to a Kirkendall-type mechanism, as suggested by the progressive cavity evolution observed in intermediate-state samples. The resulting composite exhibits outstanding EMW absorption, attributed to the synergistic effects of dielectric–magnetic losses, interfacial polarization, and multiple scattering. Compared with previously reported CoFe/CoFe_2_O_4_/carbon absorbers, the present work couples a naturally porous lotus-leaf-derived biochar scaffold with hollow CoFe/CoFe_2_O_4_ nanoparticles grown in situ via a solvothermal–pyrolysis–oxidation route: the porous framework favors uniform dispersion of the nanoparticles and multiple scattering of incident waves, while the hollow magnetic particles, together with the carbon scaffold, improve dielectric–magnetic matching.

## 2. Experimental Section

### 2.1. Materials

Cobalt acetylacetonate (Co^2+^), Iron acetylacetonate (Fe^3+^), and ammonia were purchased from Aladdin. Lotus leaves were produced in Jining, China. Additionally, 95% Ar/H_2_ gas cylinders were purchased from Kunming Peng Yi da Gas Products Co. (Kunming, China).

### 2.2. Synthesis of Biochar

In this experiment, lotus leaves were the raw material for the production of biochar. Initially, the lotus leaf samples were subjected to a three-step washing process, alternating between deionized water and ethanol to eliminate surface impurities. Subsequently, specimens underwent thermostatic desiccation at 60 °C in a precision drying chamber until achieving complete dehydration equilibrium. Following the drying process, the lotus leaf and KOH were meticulously amalgamated in accordance with a precise mass ratio, thereby facilitating the execution of chemical activation operations. The mixture was transferred to a tube furnace and heated to the pre-set temperatures of 700 °C, 800 °C, and 1000 °C at a linear heating rate of 5 °C/min in a high-purity N_2_ protective atmosphere, respectively. The constant-temperature heat treatment was carried out for 2 h at the corresponding temperatures. Subsequently, specimens were subjected to iterative deionized water rinsing to ensure complete removal of KOH residues and reaction-derived impurities. The dried specimens were processed through a forced-air drying chamber at 100 °C during a 4 h cycle, yielding a biochar series subsequently labeled as C-700, C-800, and C-1000 based on their respective pyrolysis temperatures.

### 2.3. Synthesis of CoFe/CoFe_2_O_4_/C Composites

Employing iron (III) acetylacetonate, cobalt (II) acetylacetonate, and biochar (C-800) as the fundamental reactants, the precursors of CoFe/CoFe_2_O_4_/C composites were fabricated through a solvothermal synthesis approach within an ethanol-based reaction system. A solid mixture of Fe(acac)_3_ (2 mmol), Co(acac)_2_ (1 mmol), and biochar (20 mg) was dispersed in an ethanol medium, followed by 20 min ultrasonication to ensure uniform dispersion of the particulate components. Subsequently, the mixed liquid was transferred into a 100 mL autoclave and hydrothermally treated at 100 °C for 12 h. Post-reaction, the product was filtered and freeze-dried to obtain the precursor.

**Scheme 1:**
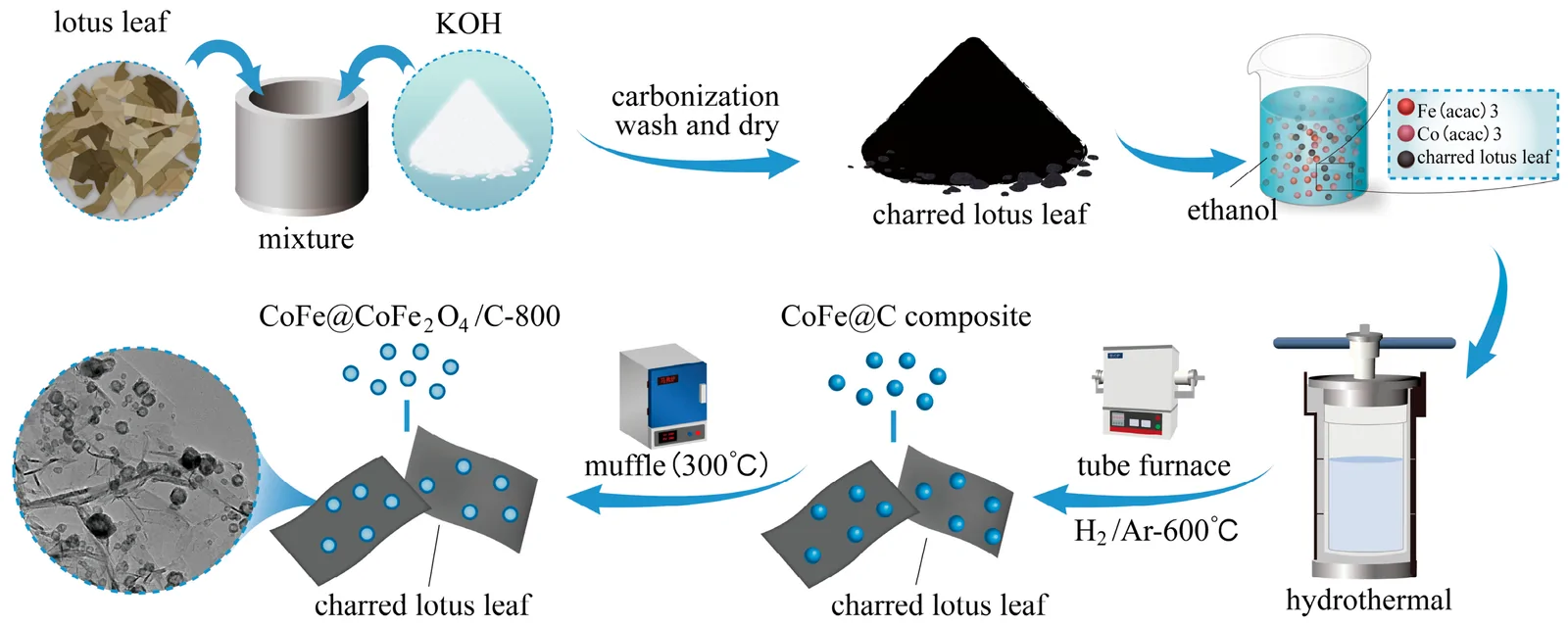
Schematic illustration of the process flow for the synthesis of CoFe/CoFe_2_O_4_/C composites.

The precursor was then positioned within a tube furnace and calcined under a controlled heating regimen (4 °C/min), ramping to 600 °C. The sample was subjected to a hydrogen–argon mixed-gas atmosphere at this temperature for a period of six hours, thereby enabling the synthesis of CoFe/C composites named CoFe/C-800. Finally, the CoFe/C composite underwent oxidative thermal processing in a muffle furnace, with programmed heating to 300 °C (4 °C/min ramp rate) under atmospheric conditions followed by a 2 h dwell time, ultimately forming the CoFe/CoFe_2_O_4_/C-800 architecture. The CoFe/CoFe_2_O_4_/C-700 composites and CoFe/CoFe_2_O_4_/C-1000 composites were prepared by exchanging biochar raw materials with different pyrolysis temperatures and following the same process flow. The process flow schematic diagram for the preparation of CoFe/CoFe_2_O_4_/C composites is shown in Scheme 1.

### 2.4. Characterizations

To comprehensively investigate the structure and properties of CoFe/CoFe_2_O_4_/C composites, various characterization methods were employed for in-depth analysis. Initially, the phase composition of the composite was analyzed by an X-ray diffractometer (XRD) (SmartLab X, Rigaku, Tokyo, Japan) with Cu Kα radiation (λ = 1.5406 Å) to ascertain its crystal structure and phase purity. Subsequently, the Raman spectrum was obtained by the 532 nm laser confocal Raman spectroscopy system (Raman) (Renishaw, In Via, Orly, France) to analyze the lattice vibration mode and chemical bond information of the material. Subsequently, X-ray photoelectron spectroscopy (XPS) (KAlpha, Thermo Fisher Scientific Co, Ltd., Waltham, MA, USA) was used to characterize the chemical valence states and surface chemical compositions of the elements in the sample. Transmission electron microscopy (TEM) (JME-2100, JEOL Ltd., Tokyo, Japan) in high-resolution mode was used to observe the microstructure, nanostructure, and lattice fringes of the composites. Meanwhile, energy-dispersive spectroscopy (EDS) was applied to conduct a quantitative analysis of the composites’ chemical composition. A physical property measurement system (PPMS) (DynalCool, Quantum Design, San Diego, CA, USA) fitted with a vibrating sample magnetometer (VSM) was utilized to test the magnetic properties of the samples at 300 K. Through this testing, relevant parameters, including the hysteresis loop, were successfully obtained. Prior to electromagnetic characterization, the composite was homogeneously blended with paraffin (1:1 mass ratio) and precision-pressed into toroidal geometries measuring 3.00 mm inner diameter and 7.00 mm outer diameter, conforming to coaxial-line measurement specifications. Altogether, the electromagnetic characteristics of the made ring samples were determined with a vector network analyzer through the coaxial approach in the 2–18 GHz frequency span. This approach was implemented to analyze the electromagnetic wave absorption characteristics of the materials.

## 3. Results and Discussion

### 3.1. Microstructure and Composition Analysis

**Figure 1:**
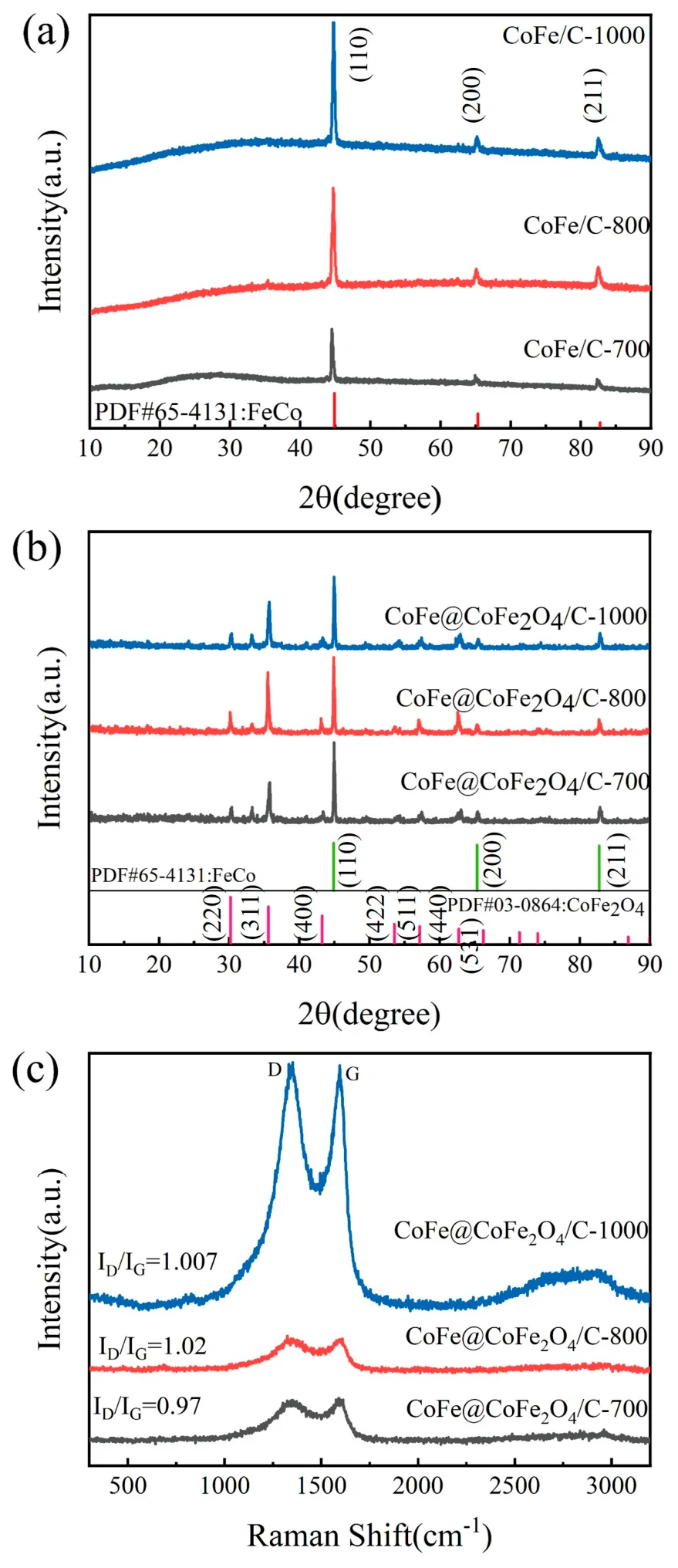
(**a**) XRD pattern of CoFe/C, (**b**) XRD pattern of CoFe/CoFe_2_O_4_/C, (**c**) Raman spectra of CoFe/CoFe_2_O_4_/C.

Figure 1a shows the XRD pattern of the sample after Ar/H_2_ heat treatment. Three distinct diffraction peaks at 44.7°, 65.3°, and 82.6° are observed, which index well to the (110), (200), and (211) planes of body-centered cubic CoFe (JCPDS No. 65-4131). No additional peaks corresponding to Fe_3_O_4_ or CoO are detected, confirming the high purity and crystallinity of the CoFe phase [23,24]. Figure 1b presents the XRD pattern after oxidation treatment. In addition to the CoFe peaks, five new peaks appear at 30.1°, 35.5°, 43.2°, 57.1°, and 62.7°, which are consistent with the Bragg reflections of CoFe_2_O_4_ (JCPDS No. 03-0864). This indicates that partial oxidation of CoFe to CoFe_2_O_4_ occurred under an air atmosphere. By controlling the annealing temperature and duration, complete oxidation was avoided; the retained CoFe phase contributes to enhanced complex permeability, which is beneficial for microwave absorption performance [25,26,27]. The crystallite sizes of the FeCo and CoFe_2_O_4_ phases were estimated from the XRD patterns in Figure 1b using the Scherrer equation (*K* = 0.89, Cu Kα radiation). For the CoFe/CoFe_2_O_4_/C-1000, CoFe/CoFe_2_O_4_/C-800, and CoFe/CoFe_2_O_4_/C-700 samples, the FeCo crystallites are approximately 39.6, 40.6, and 38.8 nm, and the CoFe_2_O_4_ crystallites are approximately 25.5, 36.1, and 31.4 nm, respectively.

Raman spectroscopy was further performed to investigate the carbon structure of the composites. As shown in Figure 1c, two characteristic bands are observed at 1345 cm^−1^ and 1588 cm^−1^, assigned to the D-band (disordered carbon/defective graphite) and G-band (graphitized carbon), respectively. The intensity ratio *I*_D_/*I*_G_ is commonly used to evaluate the degree of carbon ordering [28,29,30]. The *I*_D_ and *I*_G_ values were obtained directly from the measured D- and G-band peak intensities without peak fitting. With increasing pyrolysis temperature, the *I*_D_/*I*_G_ ratio shows a slight increase from 0.97 (CoFe@CoFe_2_O_4_/C-700) to 1.02 (CoFe@CoFe_2_O_4_/C-800), and then remains essentially unchanged at 1.007 (CoFe@CoFe_2_O_4_/C-1000). Given the small magnitude of these variations, the defect–graphitization state of the carbon scaffold is considered to be largely similar across the three samples.

X-ray photoelectron spectroscopy (XPS) was employed to investigate the surface chemical states and elemental composition of the composite. The survey spectrum (Figure S1) of the optimized CoFe/CoFe_2_O_4_/C-800 composite confirms the coexistence of Fe, Co, O, and C, consistent with the XRD and TEM results. The C 1s spectra (Figure S1a) show three characteristic peaks at 284.8, 286.2, and 288.2 eV, assigned to C–C/C=C, C–O, and C=O, respectively, following the conventional binding-energy sequence. For the Fe 2p spectra (Figure S1b), the peaks at 710.2 eV and 723.35 eV are attributed to Fe^2+^2p_3/2_ and Fe^2+^2p_1/2_. The peaks are located at 712.2 eV and 725.2 eV, which correspond to Fe^3+^ 2p_3/2_ and Fe^3+^ 2p_1/2_. The Co^2+^ 2p_1/2_ and Co^2+^ 2p_3/2_ spectra (Figure S1c) are spin-orbital-coupled, accompanied by satellite peaks. The two peaks at 781.7 and 797.1 eV correspond to Co^2+^ 2p_3/2_ and Co^2+^ 2p_1/2_ ionization, respectively. For the O 1s spectra (Figure S1d), three components are resolved: the peak at 529.7 eV is assigned to the Metal–O(lattice oxygen of CoFe_2_O_4_), that at 530.5 eV to C=O(related oxygen species), and that at 531.7 eV to C–O(adsorbed oxygen (–OH or O_2_)) [26,31,32,33]. These results confirm the coexistence of Fe^2+^/Fe^3+^ and Co^2+^ species, further supporting the formation of CoFe alloy and CoFe_2_O_4_ phases in the composite.

**Figure 2:**
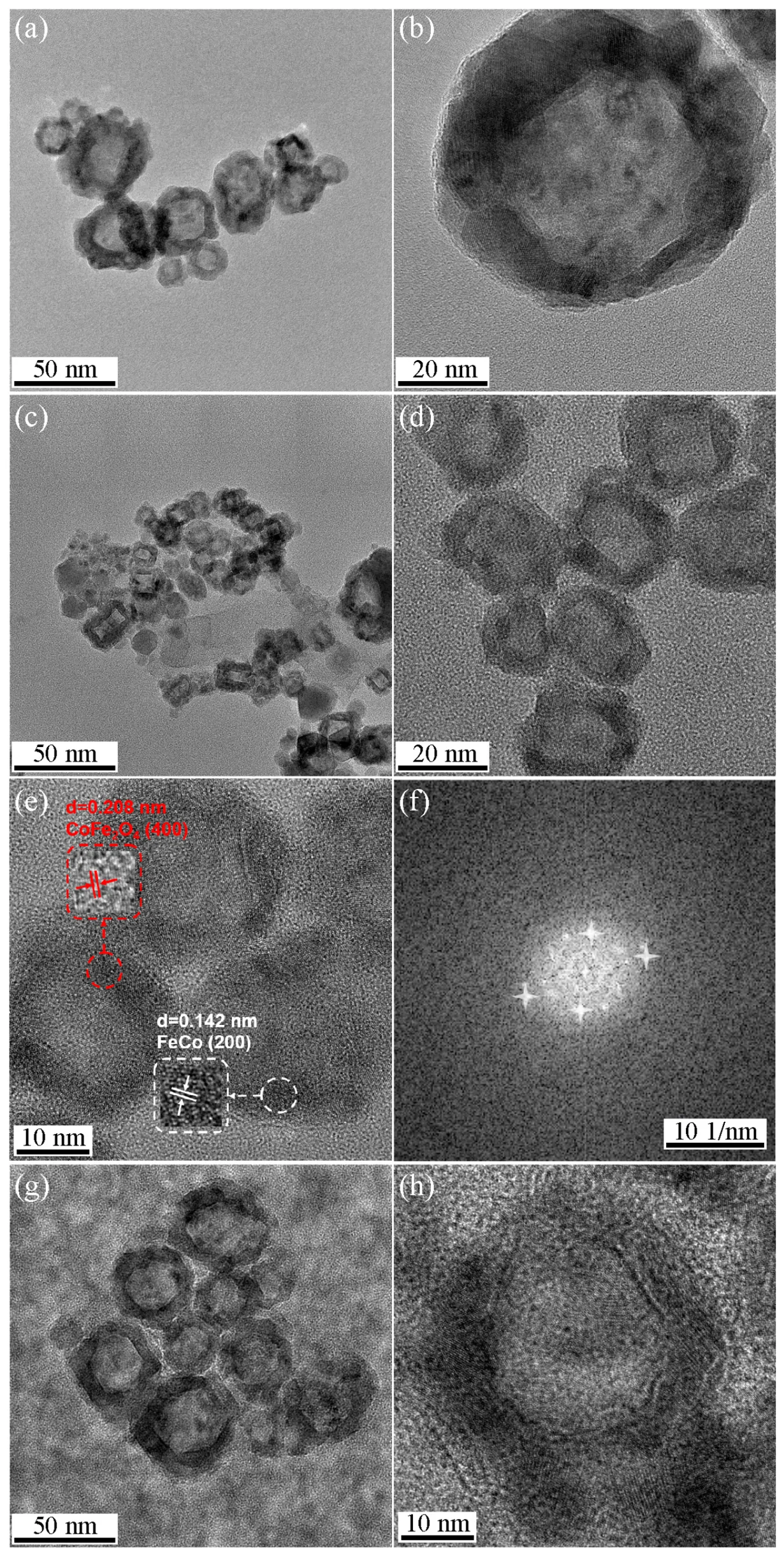
(**a**,**b**) TEM images of CoFe/CoFe_2_O_4_/C-700, (**c**,**d**) TEM images of CoFe/CoFe_2_O_4_/C-800, (**e**) HRTEM images of CoFe/CoFe_2_O_4_/C-800, (**f**) the FFT pattern derived from the HRTEM image, (**g**,**h**) TEM images of CoFe/CoFe_2_O_4_/C-1000.

Microstructural features including morphology, crystallographic lattice, and elemental distribution were resolved through TEM/HRTEM imaging coupled with EDS analysis. Figure 2a,b show the morphological characteristics of CoFe/CoFe_2_O_4_/C-700. The microscopic images distinctly show that the material has a classic hollow nanostructure. With a wide particle size distribution in the range of 5–20 nm, the thickness of the particle walls remains relatively uniform at about 2 nm. It is worth noting that there is obvious size heterogeneity among different particles, which may be related to the shrinkage behavior of the carbon matrix and the difference in the diffusion rate of metal precursors during pyrolysis [34,35].

Figure 2c,d depict the microstructure evolution process of CoFe/CoFe_2_O_4_/C-800. Compared with the 700 °C sample, the nanoparticles treated at 800 °C showed a more uniform dispersion state on the biochar carrier; the particle size distribution was significantly narrowed to about 20 nm, while maintaining a stable wall thickness of 2 nm. Notably, the distinctive hollow rectangular structure is seen for the first time in Figure 2d. The reason behind this phenomenon can be traced back to the kinetic effect during the oxidation process: the selective oxidation of iron leads to local lattice reconstruction, combined with the volume expansion/contraction effect caused by thermal stress, and finally forms a hollow structure with geometric symmetry. Figure 2e,f illustrate the further analysis of the crystal structure of CoFe/CoFe_2_O_4_/C-800, which was conducted by high-resolution transmission electron microscopy. Figure 2e shows that the material has obvious lattice heterojunction characteristics: the regular lattice fringe of 0.142 nm corresponds to the (200) crystal plane of the metal alloy CoFe, while the shallow fringe of 0.208 nm is perfectly matched with the (400) crystal plane of spinel CoFe_2_O_4_. These observations, together with the XRD results, indicate that the CoFe and CoFe_2_O_4_ phases coexist and are intimately mixed within the hollow nanoparticles [36,37,38].

From Figure 2g,h, it can be seen that upon increasing the pyrolysis temperature to 1000 °C, CoFe/CoFe_2_O_4_/C-1000 still sustains the core framework of the hollow structure. Yet, there is an observable tendency for the particles to aggregate, and the distribution expands further. This may be caused by the dissolution of small particles on the biochar surface. As opposed to the low-temperature samples, the particle size distribution expands further. This may be caused by the dissolution of small particles and the promotion of large particle growth, which are brought about by the Ostwald ripening process occurring at elevated temperatures.

To further examine the cavity formation, intermediate-state samples of CoFe/CoFe_2_O_4_/C-800 obtained at different oxidation times were observed by TEM (Figure S2). A progressive evolution from solid particles to partially hollow and finally fully hollow structures is observed, which supports a Kirkendall-type formation mechanism.

**Figure 3:**
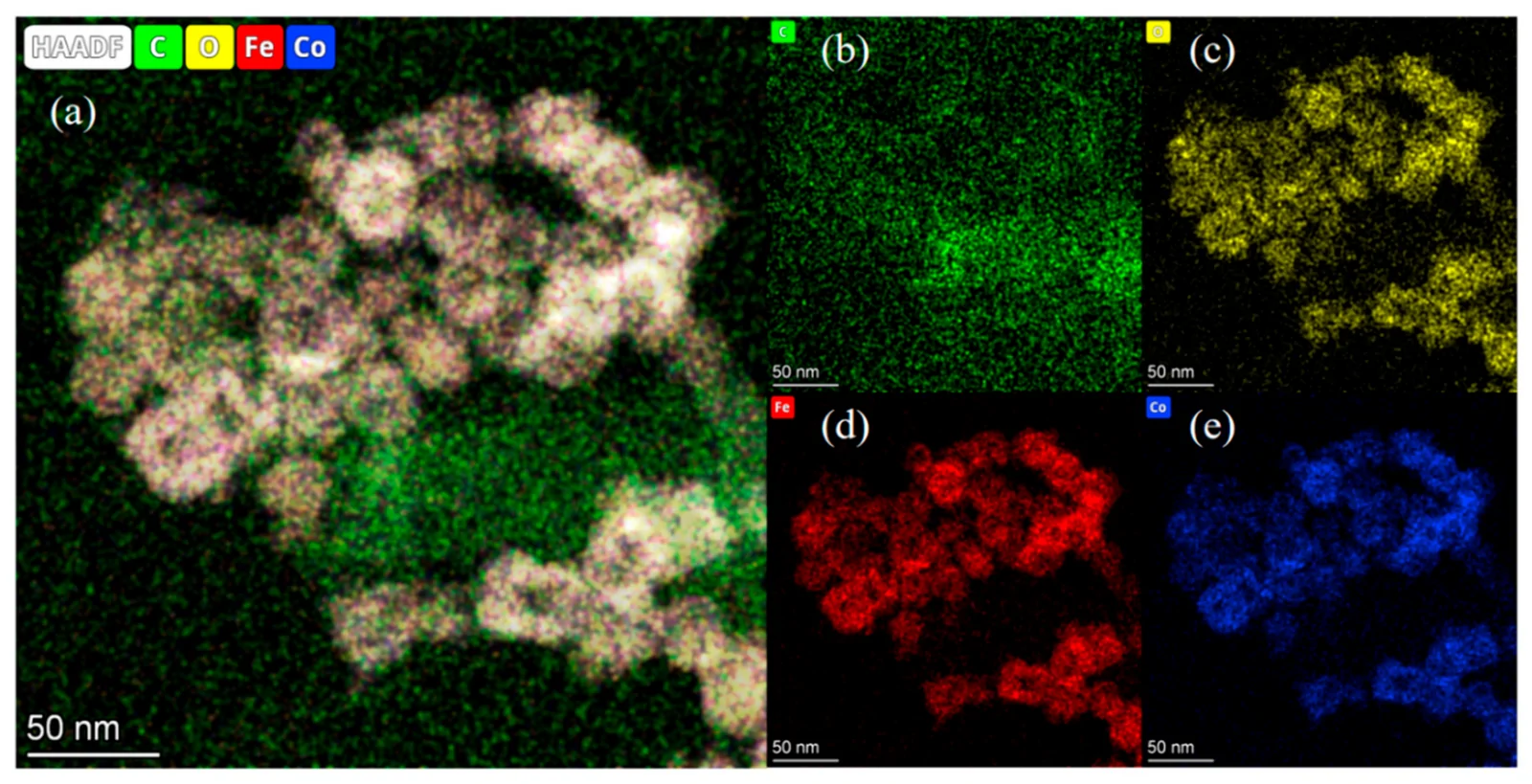
Energy-dispersive X-ray (EDS) analysis for elemental mapping. Images (**a**–**e**) correspond to different elemental mapping images of CoFe/CoFe_2_O_4_/C-800 composites, respectively.

As shown in the mapping diagram of Figure 3a, the green color indicates carbon elements (Figure 3b), the yellow color represents oxygen elements (Figure 3c), the red color stands for iron elements (Figure 3d), and the blue color denotes cobalt elements (Figure 3e). Clearly, carbon is located at the base, and iron, cobalt, and oxygen combine to form CoFe/CoFe_2_O_4_ particles distributed on the carbon. Moreover, this finding, along with the results from XRD and XPS characterizations, jointly confirms the presence of Co, Fe, and O elements [39,40]. The atomic contents of Fe, Co, and O in the probed region were 40.5, 17.2, and 42.3 at%, respectively. The carbon content is not quantified because the TEM specimens were prepared on carbon-coated grids.

The pore structure of CoFe/CoFe_2_O_4_/C-800 was characterized by N_2_ adsorption–desorption measurements, and the results are shown in Figure S3. As shown in Figure S3b, the N_2_ adsorption–desorption isotherm of CoFe/CoFe_2_O_4_/C-800 exhibits a sharp uptake at low relative pressure (*P*/*P*_0_ < 0.1), characteristic of a typical type I isotherm, indicating that the material is predominantly microporous. The subsequent steep rise at high relative pressure (*P*/*P*_0_ > 0.9), accompanied by a small hysteresis loop, suggests the presence of a small fraction of meso-/macropores. The corresponding pore size distribution (Figure S3a) shows two sharp peaks at around 10 Å, confirming the abundance of uniform micropores, together with a broad continuous distribution in the range of 20–50 Å, reflecting a hierarchical micro-mesoporous architecture. The BET specific surface area was calculated from the linear region of the BET plot (*P*/*P*_0_ = 0.05–0.35, R^2^ = 0.999); this range was selected because it satisfies the BET consistency criteria (positive intercept, monotonically increasing adsorption, and a positive BET C constant ≈ 80), which is physically reasonable for a predominantly microporous carbon. The resulting BET surface area is 141 m^2^/g. The total pore volume, estimated from the single-point adsorption at *P*/*P*_0_ ≈ 0.99, is 0.20 cm^3^/g.

### 3.2. Magnetic Analysis

**Figure 4:**
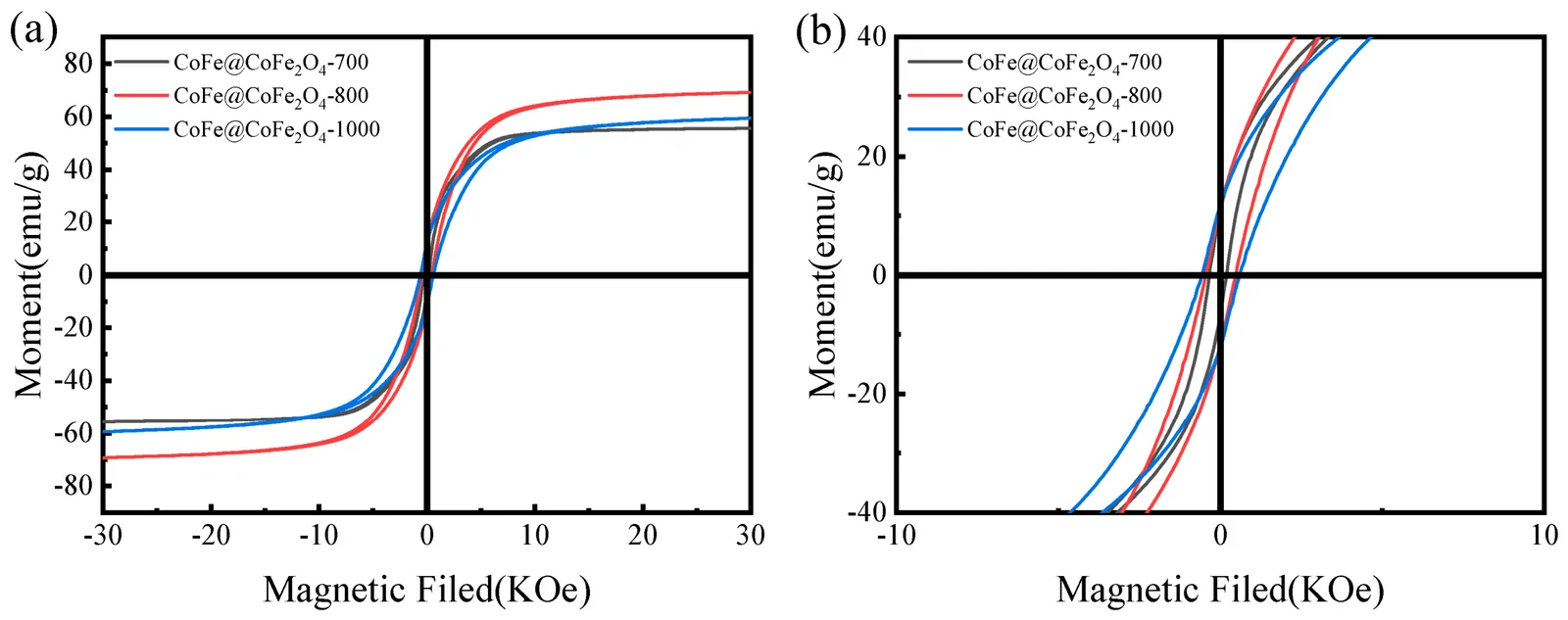
(**a**) Magnetic hysteresis loops and (**b**) partial enlarged view of CoFe/CoFe_2_O_4_/C.

Figure 4 shows the hysteresis loops for the corresponding saturation magnetization intensity (*M*_s_) and coercivity (*H*_c_). It can be found that CoFe/CoFe_2_O_4_/C-700, 800, and 1000 have maximum saturation magnetization strengths of 55.62 emu/g, 69.20 emu/g, and 59.40 emu/g, respectively, and the coercivity is 193 Oe, 482 Oe, and 658 Oe, respectively. Among the three samples, CoFe/CoFe_2_O_4_/C-800 exhibits the highest *M*_s_ (69.20 emu/g). The *M*_s_ of a magnetic composite is governed by multiple factors—including phase composition, crystallinity, particle size and dispersion, and magnetic loading—and the individual contributions of these factors have not been quantitatively separated in the present study. C-800 shows the strongest and sharpest XRD peaks and uniformly dispersed nanoparticles, whereas C-700 shows relatively weak XRD peaks and finer particles, and C-1000 retains high CoFe-phase peak intensities but exhibits more pronounced particle growth and aggregation. These microstructural differences may collectively contribute to the observed variation in *M*_s_ [41,42,43,44]. In addition, the combination of low coercivity and high saturation magnetization in all samples provides a critical foundation for optimizing their magnetic loss performance [45].

### 3.3. Electromagnetic Wave Absorption

The electromagnetic parameters, complex permittivity (*ε* = *ε*′ − *jε*″), and permeability (*μ* = *μ*′ − *jμ*″) directly determine the EMW absorption performance. The real parts (*ε*′, *μ*′) reflect the storage capacity of electric and magnetic energy, while the imaginary parts (*ε*″, *μ*″) represent the dissipation from dielectric and magnetic losses. Higher *ε*″ and *μ*″ values indicate enhanced polarization relaxation, conductive loss, or magnetic resonance, facilitating electromagnetic-to-thermal energy conversion [46,47]. In addition to intrinsic loss capability, impedance matching and the quarter-wavelength cancelation condition are critical for achieving optimal absorption, as described by transmission line theory. Specifically, dielectric loss arises from interfacial polarization, dipole polarization, and conduction loss, whereas magnetic loss originates from natural resonance, exchange resonance, and eddy current effects [48].

The microstructural features are also expected to contribute to the electromagnetic responses. Specifically, the hollow cavities and thin shells may reduce the effective density of the magnetic particles and prolong the propagation path of incident waves, favoring internal multiple reflections; the abundant heterointerfaces (CoFe/CoFe_2_O_4_, CoFe_2_O_4_/C, C/air) may act as additional interfacial-polarization sites that enhance the dielectric response; and the porous biochar scaffold with well-dispersed nanoparticles is likely to form an extended conduction network and promote multiple scattering.

**Figure 5:**
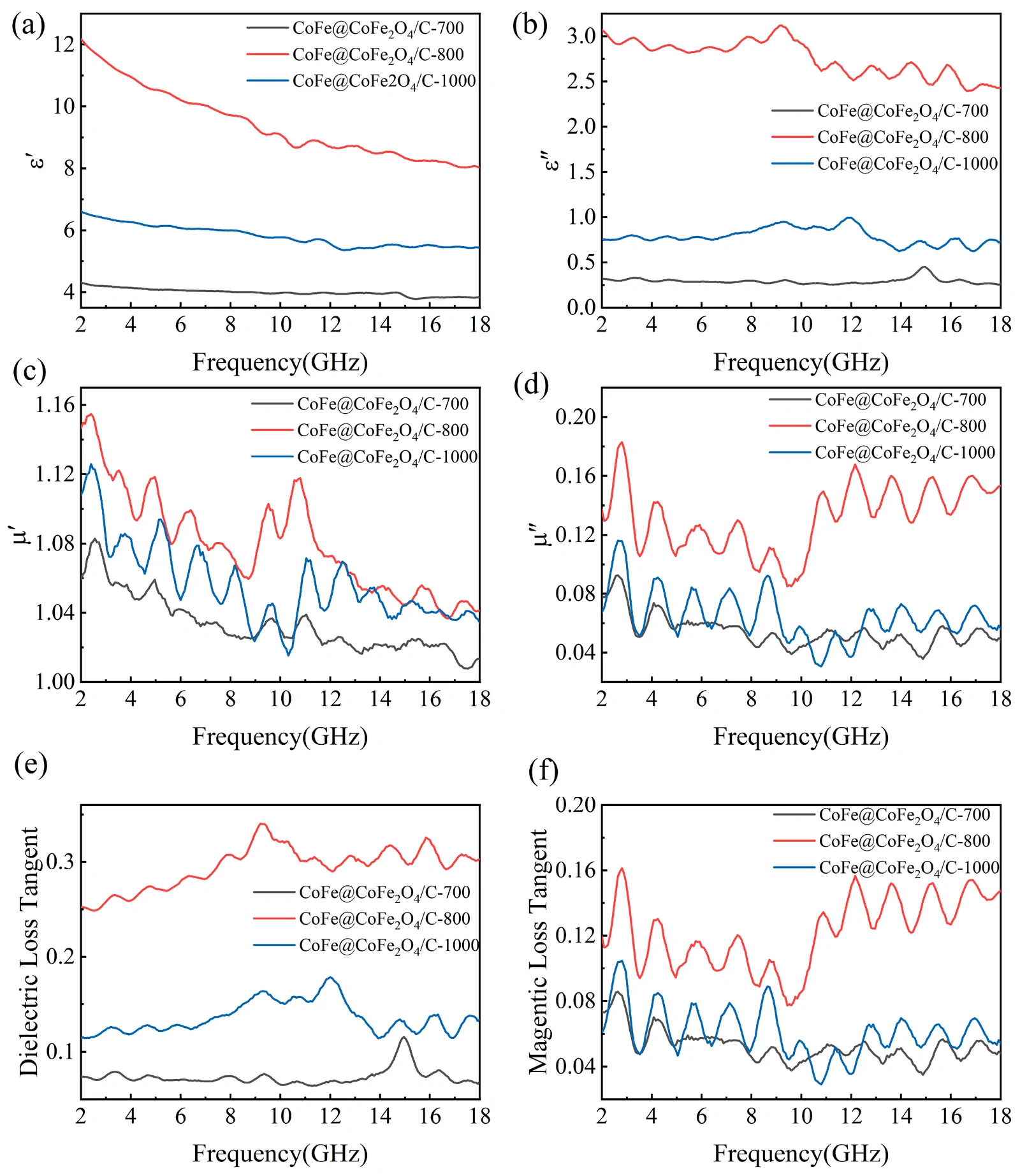
(**a**) Real part of permittivity (*ε*′); (**b**) imaginary part of permittivity (*ε*″); (**c**) real part of permeability (*μ*′); (**d**) imaginary part of permeability (*μ*″); (**e**) dielectric loss tangent (tan*δε*); (**f**) magnetic loss tangent (tan*δμ*).

Figure 5a reveals a frequency-dependent decline in the real permittivity (*ε*′) of the CoFe/CoFe_2_O_4_/C composite, consistent with interfacial polarization relaxation dynamics in heterogeneous systems. The *ε*′ of the CoFe@CoFe_2_O_4_/C-700, CoFe/CoFe_2_O_4_/C-800, and CoFe/CoFe_2_O_4_/C-1000 composites are 4.30–3.78, 12.17–8.01, and 6.59–5.34, respectively. Figure 5b demonstrates oscillatory behavior in the imaginary permittivity (*ε*″) of the CoFe/CoFe_2_O_4_/C composite across the frequency spectrum, indicative of polarization relaxation dynamics at heterogeneous interfaces. The *ε*″ values of CoFe/CoFe_2_O_4_/C-700, CoFe/CoFe_2_O_4_/C-800 and CoFe/CoFe_2_O_4_/C-1000 composites are 0.45–0.26, 3.12–2.42 and 0.99–0.62, respectively. Both *ε*′ and *ε*″ exhibited an initial rise followed by a decline with elevated biochar pyrolysis temperatures. As the defect–graphitization state is broadly similar among the three samples, the *ε*″ differences are likely related to differences in polarization relaxation intensity rather than to differences in graphitization. A prominent feature emerges in the *ε*″ spectra across all specimens, with multiple relaxation signatures detected, signifying heterogeneous polarization dynamics. In terms of the mechanism of action, analysis can be conducted from two distinct perspectives. Firstly, the internal heterogeneous interface structure of the material constructs an effective separation interface between CoFe/CoFe_2_O_4_ nanoparticles and the carbon-based phase. The interfacial domains effectively confine charge carriers migrating under electromagnetic excitation, generating pronounced interfacial polarization that enhances the material’s dielectric attenuation efficiency. Secondly, the presence of functional groups and structural imperfections within the graphitized carbon area offers the structural underpinnings for the development of dipole polarization centers. It has been demonstrated that the dielectric loss of dipoles in an alternating electric field exhibits a dispersion fluctuation behavior with frequency [49,50]. From Figure 5c, it can be seen that the *μ*′ of CoFe/CoFe_2_O_4_/C decreases with the increase in frequency. The *μ*′ of CoFe/CoFe_2_O_4_/C-700, CoFe/CoFe_2_O_4_/C-800, and CoFe/CoFe_2_O_4_/C-1000 composites are 1.08–1.01, 1.15–1.04, and 1.12–1.01, respectively. Figure 5d reveals oscillatory *μ*″ behavior in CoFe@CoFe_2_O_4_/C, reflecting dynamic magnetic relaxation and eddy current effects across the frequency domain. The *μ*″ values of CoFe/CoFe_2_O_4_/C-700, CoFe/CoFe_2_O_4_/C-800, and CoFe/CoFe_2_O_4_/C-1000 composites are 0.09–0.03, 0.18–0.08, and 0.11–0.03, respectively. Pronounced resonance signatures and oscillatory features are observed in the *μ*′ and *μ*″ spectra. These features may involve multiple magnetic relaxation mechanisms, such as natural ferromagnetic resonance, nanoscale confinement, enhanced surface anisotropy, and spin-wave excitations inherent to spinel-structured CoFe_2_O_4_ nanoparticles [51,52]. Based on the previous analysis, the dielectric loss tangent (tanδ*_ε_* = *ε*″/*ε*′) and the magnetic loss tangent (tanδ*_μ_* = *μ*″/*μ*′) are then calculated, as illustrated in Figure 5e,f. It can be seen that the two types of loss tangents show complex dispersion characteristics with slight fluctuations, and the trends of the two changes are significantly opposite, indicating that the dielectric loss and magnetic loss show good complementary characteristics in this frequency range.

**Figure 6:**
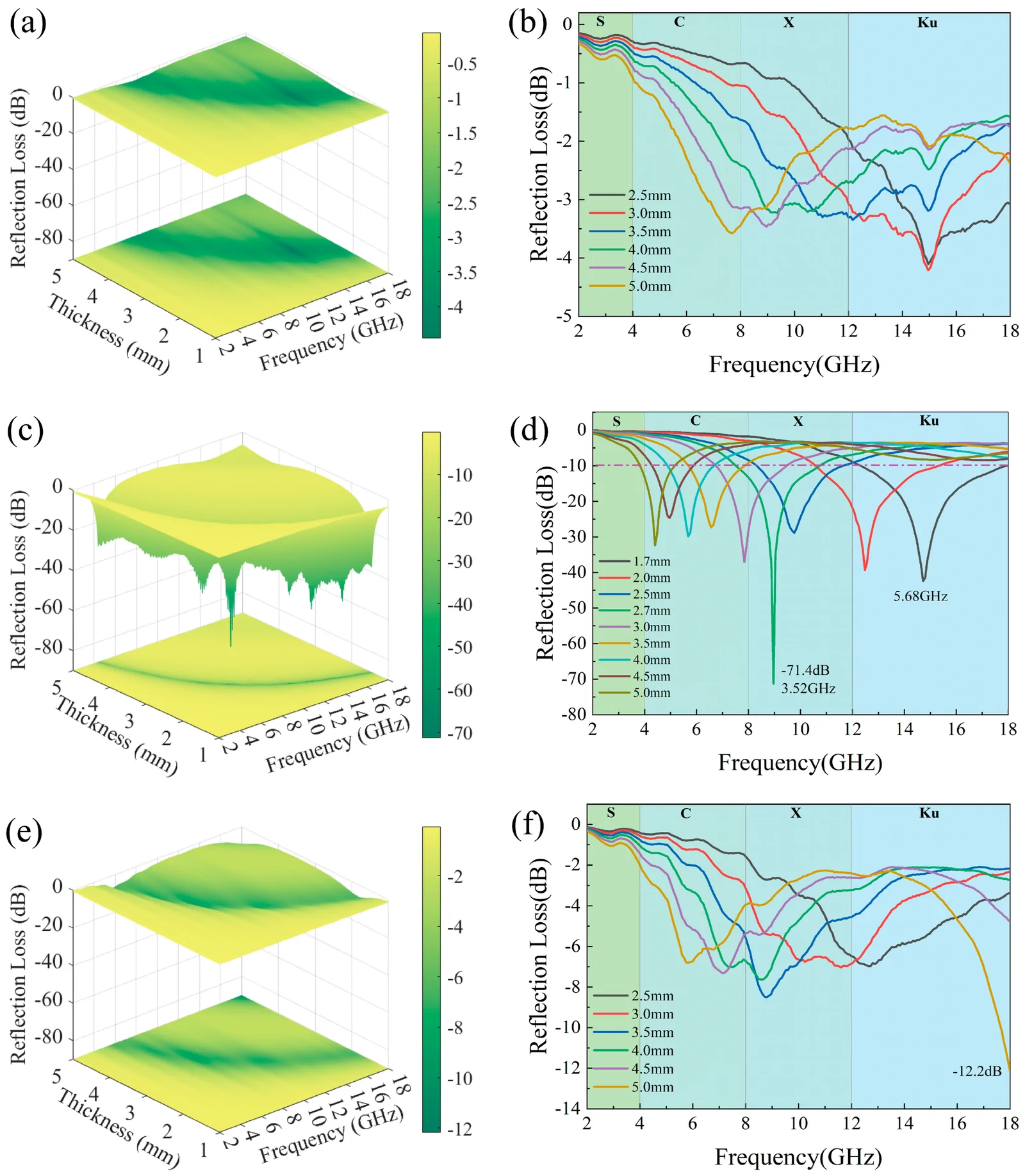
Three-dimensional and two-dimensional *RL* values of composite materials CoFe/CoFe_2_O_4_/C-700 (**a**,**b**), CoFe/CoFe_2_O_4_/C-800 (**c**,**d**), CoFe/CoFe_2_O_4_/C-1000 (**e**,**f**).

The electromagnetic wave absorption performance of the prepared samples was evaluated using transmission line theory. The reflection loss (*RL*) is calculated by the following equation [53]:

(1) $$\mathrm{RL}(\mathrm{dB})=20\log\left|\frac{Z_{\mathrm{in}}-Z_{0}}{Z_{\mathrm{in}}+Z_{0}}\right|$$

(2) $$Z_{\mathrm{in}}=Z_{0}\sqrt{\frac{\mu_{r}}{\varepsilon_{r}}}\tanh\left[j(\frac{2\mathrm{\pi fd}}{c})\sqrt{\mu_{r}\varepsilon_{r}}\right]$$

where *Z*_in_ and *Z*_0_ represent the normalized input impedance and free-space impedance of the composite material, respectively. The frequency of the incident electromagnetic wave is denoted by *f*, the thickness of the absorbing material by *d*, the speed of vacuum light by *c*, and the complex permittivity and complex permeability of the composite material by *ε_r_* and *μ_r_*, respectively. Figure 6 presents three-dimensional and two-dimensional *RL* values of composite materials CoFe/CoFe_2_O_4_/C-700 (a, b), CoFe/CoFe_2_O_4_/C-800 (c, d) and CoFe/CoFe_2_O_4_/C-1000 (e, f). All *RL* values were calculated from the measured complex permittivity and permeability using transmission line theory. Among them, CoFe/CoFe_2_O_4_/C-800 (Figure 6c,d) exhibits the best EMW absorption performance. At a matching thickness of 2.7 mm, the minimum reflection loss (*RL*_min_) reaches −71.4 dB at 8.96 GHz. When the thickness is reduced to 1.7 mm, the effective absorption bandwidth (EAB) extends to 5.68 GHz, covering most of the Ku band. In contrast, CoFe/CoFe_2_O_4_/C-700 and CoFe/CoFe_2_O_4_/C-1000 show much inferior performance, with *RL*_min_ values of only −4.21 dB at 3.0 mm and −12.2 dB at 5.0 mm, respectively.

**Figure 7:**
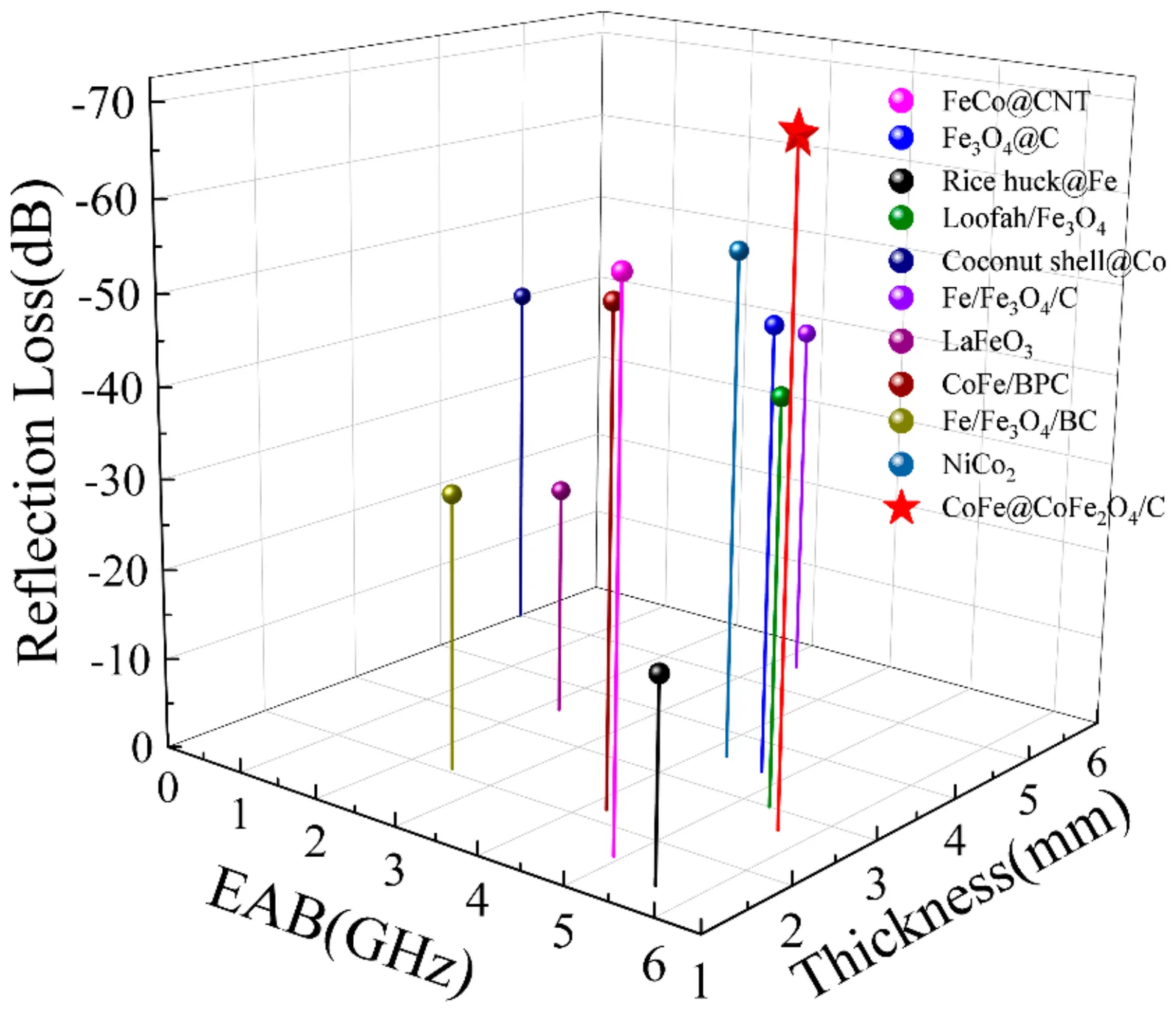
Comparison of *RL*_min_ values, thickness, and EAB for reported microwave-absorbing materials.

As shown in Figure 7, compared with previously reported magnetic metal and carbon-based absorbers, the CoFe/CoFe_2_O_4_/C-800 composite achieves a favorable combination of strong absorption and broad bandwidth at low thickness and density. These results demonstrate its potential as a high-performance EMW absorbing material. The specific parameters of the materials presented in Figure 7 are listed in Table 1.

According to the quarter-wavelength matching model, maximum absorption occurs at the frequency where the absorber thickness tm satisfies the following equation [60]:

(3) $$t_{m}=\frac{\mathrm{n\lambda}}{4}=\frac{\mathrm{nc}}{4f_{m}{\left(\left|\varepsilon_{r}\right|\left|\mu_{r}\right|\right)}^{\frac{1}{2}}}\;\;\;\;n=1,3,5\ldots$$

The calculated RL peaks of CoFe@CoFe_2_O_4_/C-800 fall closely on the *λ*/4 curve, indicating that its thickness satisfies the quarter-wavelength condition. Under this condition, the phase difference between the incident and reflected waves at the air–absorber interface is 180°, resulting in destructive interference that minimizes reflection and enhances absorption [49]. In contrast, the calculated data for CoFe/CoFe_2_O_4_/C-700 and CoFe/CoFe_2_O_4_/C-1000 deviate significantly from the curve, consistent with their inferior absorption performance. Thus, the excellent EMW absorption of CoFe/CoFe_2_O_4_/C-800 can be partly attributed to the proper alignment of absorber thickness with the quarter-wavelength condition.

The impedance matching characteristic (*Z*) is crucial for minimizing electromagnetic wave reflection. When the material’s normalized impedance *Z*_in_ equals 1 (|*Z*_in_ − 1| = 0), the material impedance matches the free-space impedance perfectly, allowing electromagnetic waves to enter without reflection. To eliminate reflection at the air–material interface, the material’s impedance should closely approximate that of free space. The relevant formula is shown below [16].

(4) $$Z=\left|\frac{Z_{\mathrm{in}}}{Z_{0}}\right|=\left|\sqrt{\frac{\mu_{r}}{\varepsilon_{r}}}\tanh\left[j(\frac{2\mathrm{\pi fd}}{c})\sqrt{\mu_{r}\varepsilon_{r}}\right]\right|$$

As shown in Figure S4, the |*Z*_in_ − 1| values corresponding to the RL peaks of CoFe/CoFe_2_O_4_/C-800 are close to zero, confirming good impedance matching. By contrast, CoFe/CoFe_2_O_4_/C-700 and CoFe/CoFe_2_O_4_/C-1000 exhibit much larger |*Z*_in_ − 1| values, indicating poor impedance matching. Overall, the outstanding absorption performance of CoFe/CoFe_2_O_4_/C-800 arises from the synergistic effects of optimized impedance matching and quarter-wavelength interference [17].

**Figure 8:**
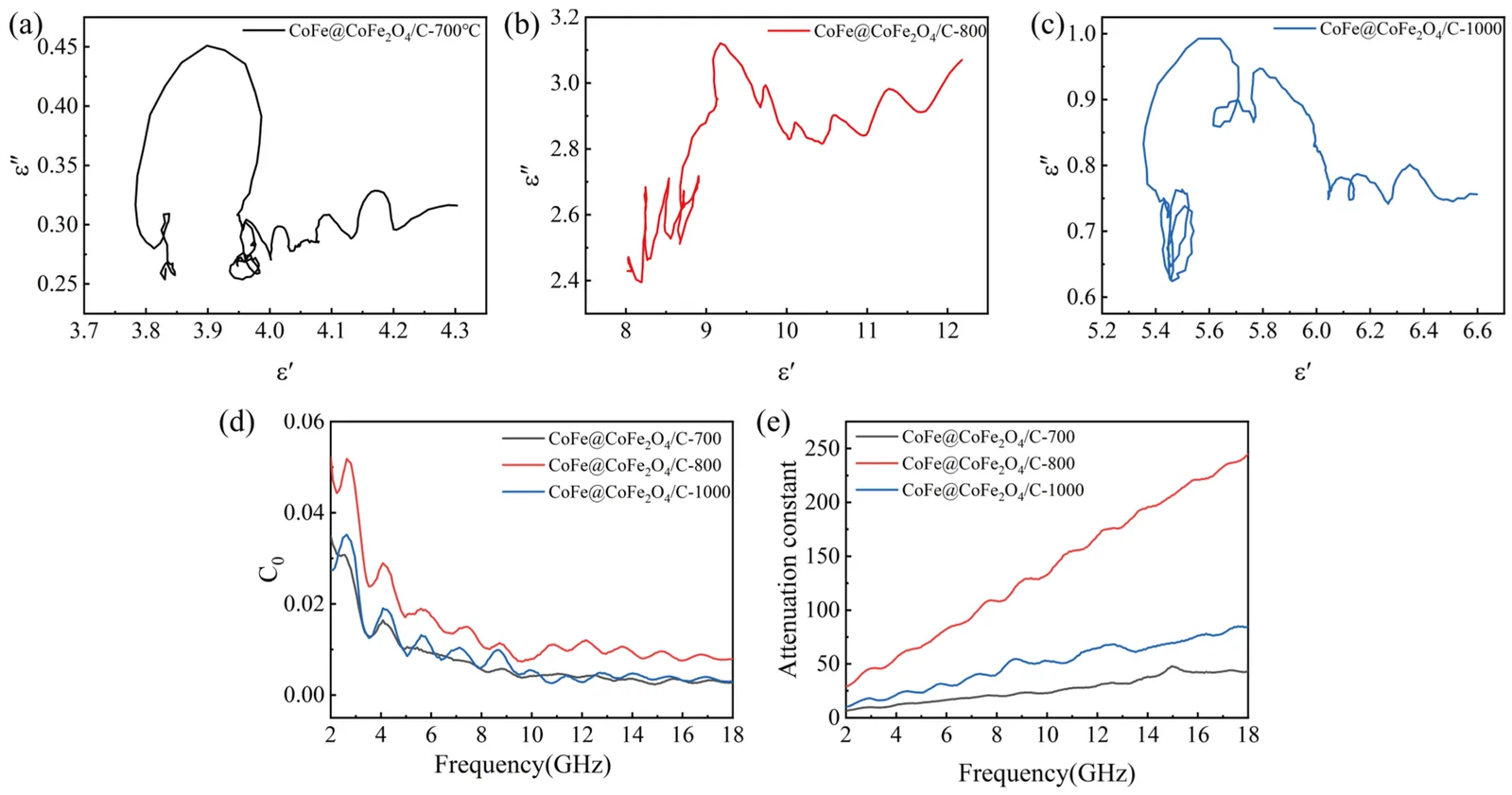
The Cole–Cole plot of (**a**) CoFe/CoFe_2_O_4_/C-700, (**b**) CoFe/CoFe_2_O_4_/C-800, (**c**) CoFe/CoFe_2_O_4_/C-1000; (**d**) the *C*_0_ values and (**e**) attenuation constant (*α*) of CoFe/CoFe_2_O_4_/C.

Dielectric loss plays a pivotal role in affecting the electromagnetic wave attenuation of composite materials. It mainly comprises physical mechanisms such as Debye relaxation, conduction loss, interface polarization, and dipole polarization. Relying on the Debye relaxation model, the Cole–Cole circle serves to visualize every polarization relaxation process, underscoring the contribution of dielectric loss to the material’s microwave absorption ability. The Debye relaxation equation is shown below [37]:

(5) $$\varepsilon^{\prime }=\varepsilon_{\infty }+\frac{\varepsilon_{s}-\varepsilon_{\infty }}{1+{\left(2\mathrm{\pi f}\right)}^{2}\tau^{2}}$$

(6) $$\varepsilon^{\prime \prime }=\varepsilon_{\infty }+\frac{2\mathrm{\pi f\tau}\left(\varepsilon_{s}-\varepsilon_{\infty }\right)}{1+{\left(2\mathrm{\pi f}\right)}^{2}\tau^{2}}$$

(7) $${\left(\varepsilon^{\prime }-\frac{\varepsilon_{s}+\varepsilon_{\infty }}{2}\right)}^{2}+{\left(\varepsilon^{\prime \prime }\right)}^{2}={\left(\frac{\varepsilon_{s}-\varepsilon_{\infty }}{2}\right)}^{2}$$

where *ε_s_* and *ε_∞_* represent the static and relative permittivity in the high frequency range, respectively. Under ideal Debye relaxation, the plot of *ε*′ versus *ε*″ yields a semicircular arc. Deviation from the ideal semicircle indicates the presence of multiple relaxation processes or additional loss mechanisms. For instance, multiple arcs suggest multiple Debye relaxations, whereas a linear tail at low frequencies indicates conductive loss. Figure 8a–c show the Cole–Cole plots of CoFe/CoFe_2_O_4_/C-700, C-800, and C-1000. All three composites exhibit deformed semicircles accompanied by low-frequency linear tails, which is consistent with the coexistence of multiple polarization relaxations and a conduction-loss contribution. Notably, among various polarization mechanisms, electronic and ionic polarizations occur predominantly at frequencies above 10^3^–10^6^ GHz and are therefore negligible in the 2–18 GHz range. Within this frequency band, interfacial and dipolar polarizations dominate. The former arises from charge accumulation at heterojunction interfaces (e.g., CoFe/CoFe_2_O_4_, CoFe_2_O_4_/C, C/air), while the latter originates from the relaxation of dipole moments associated with functional groups and structural defects [17].

Magnetic loss is another critical contributor to EMW attenuation. Theoretically, it encompasses domain wall resonance, natural resonance, eddy current loss, and hysteresis loss. Since domain wall resonance typically occurs in the MHz range, its contribution is negligible in the 2–18 GHz band. Hysteresis loss is also excluded because it only appears under strong magnetic fields, whereas the magnetic field of EMW is weak. The eddy current loss can be evaluated by the *C*_0_ parameter, which is expressed as [8]:

(8) $$C_{0}=\mu^{\prime \prime }{\left(\mu^{\prime }\right)}^{-2}f^{-1}$$

If eddy current loss dominates, *C*_0_ should remain approximately constant over the test frequency range. However, as shown in Figure 8d, the *C*_0_ values of all three composites vary significantly with frequency, indicating that eddy current loss is not the primary magnetic loss mechanism. Beyond eddy current loss, other magnetic loss mechanisms such as natural resonance are expected to contribute [45]. Surface anisotropy and interfacial exchange coupling between the FeCo alloy and CoFe_2_O_4_ are considered plausible contributors to the enhanced magnetic response. The *C*_0_ value of CoFe/CoFe_2_O_4_/C-800 exhibits more pronounced frequency-dependent fluctuations than those of the other two samples, suggesting a stronger magnetic loss response.

The overall attenuation performance is further evaluated by the attenuation constant (*α*), calculated as follows [32]:

(9) $$\alpha =\frac{\sqrt{2}\mathrm{\pi f}}{c}\sqrt{\left(\mu^{\prime \prime }\varepsilon^{\prime \prime }-\mu^{\prime }\varepsilon^{\prime }\right)+\sqrt{{\left(\mu^{\prime \prime }\varepsilon^{\prime \prime }-\mu^{\prime }\varepsilon^{\prime }\right)}^{2}+{\left(\mu^{\prime \prime }\varepsilon^{\prime \prime }+\mu^{\prime }\varepsilon^{\prime }\right)}^{2}}}$$

As shown in Figure 8e, CoFe/CoFe_2_O_4_/C-800 exhibits the highest *α* values across the entire 2-18 GHz range, confirming its superior attenuation capability. This enhancement is attributed to the increased *ε*” and *μ*”, which promote energy dissipation [26]. However, excessive optimization of electromagnetic parameters may degrade impedance matching: when *ε*” and *μ*” exceed critical limits, the input impedance deviates from that of free space, creating a reflective interface that prevents incident waves from entering the absorber. Therefore, achieving optimal absorption requires a balanced design that simultaneously ensures strong attenuation and good impedance matching through rational component and structural engineering.

### 3.4. RCS Simulation

To evaluate the practical EMW absorption performance, a double-layer square radar cross-section (RCS) model was constructed in CST Studio Suite. The lower layer was set as a perfect electric conductor (PEC), and the upper layer was the absorbing material with a thickness of 2.7 mm, matching the quarter-wavelength condition at 8.96 GHz. The dielectric constant and permeability at this frequency were assigned to the absorber layer. The simulation employed an open boundary in the Z direction and a plane wave excitation along the X direction. A far-field monitor was defined at the target frequency, and the incident angle *θ* was swept from −90° to 90° using the parameter scanning tool to extract monostatic RCS data.

**Figure 9:**
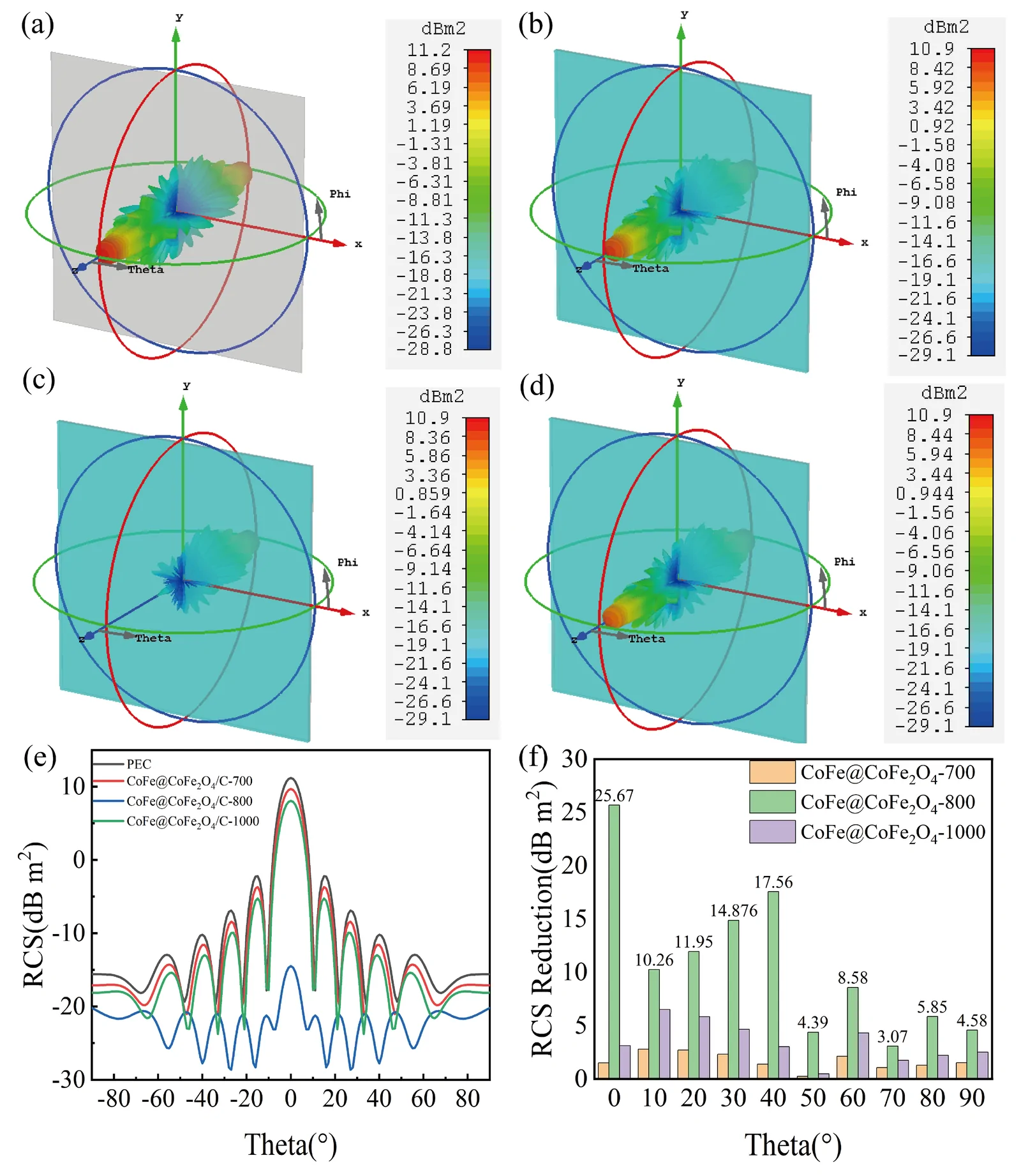
CST simulation results of the samples: (**a**) perfect conductive layer (PEC) and the perfect conductive layer covered with (**b**) CoFe/CoFe_2_O_4_/C-700, (**c**) CoFe/CoFe_2_O_4_/C-800, and (**d**) CoFe/CoFe_2_O_4_/C-1000 samples. (**e**) RCS simulated curves of PEC, CoFe/CoFe_2_O_4_/C different scanning angles. (**f**) Comparison of the RCS reduction values of CoFe/CoFe_2_O_4_/C samples.

Figure 9a–d present the simulated RCS results. The bare PEC substrate exhibits the strongest backscattering. The CoFe/CoFe_2_O_4_/C-700/PEC and CoFe/CoFe_2_O_4_/C-1000/PEC structures show similar scattering behavior to the bare PEC, with only a slight reduction observed for the latter in certain frequency bands. In contrast, the CoFe/CoFe_2_O_4_/C-800/PEC structure demonstrates significantly enhanced stealth performance, with RCS values more than 15 dB·m^2^ lower than those of the PEC substrate across all angles, confirming its superior impedance matching characteristics.

The angular RCS curves (Figure 9e,f) reveal typical specular reflection at normal incidence (*θ* = 0°). As *θ* deviates toward ±90°, the RCS gradually decreases, accompanied by periodic oscillations arising from the competition between edge diffraction and surface wave coupling. Notably, the CoFe/CoFe_2_O_4_/C-800/PEC structure maintains an RCS below −10 dB·m^2^ over the entire wide-angle range (θ = 0–±90°), indicating that the heterogeneous interface effectively suppresses multipath scattering. Specifically, the RCS reductions (ΔRCS = RCS _PEC_ − RCS _sample_) at scattering angles of 0°, 10°, 20°, 30°, and 40° are 25.67, 10.26, 11.95, 14.87, and 17.56 dB·m^2^, respectively. The maximum reduction at normal incidence (*θ* = 0°) further confirms the optimal impedance matching at vertical incidence. The simulation predicts that the CoFe/CoFe_2_O_4_/C-800 composite exhibits exceptional wide-angle electromagnetic scattering suppression capability, suggesting potential for radar-stealth-related applications.

### 3.5. EMW Absorption Mechanism

**Scheme 2:**
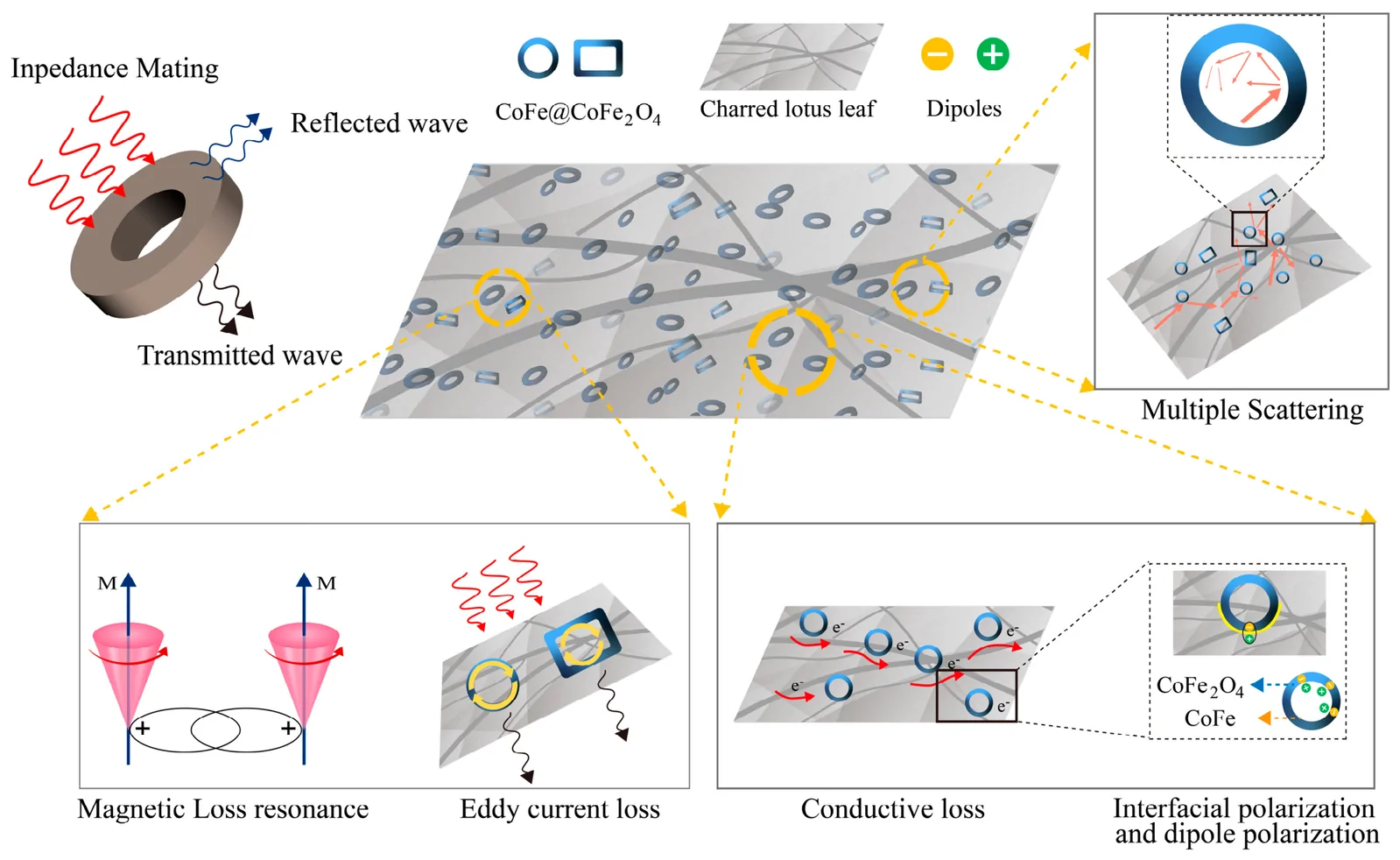
The schematic chart of the proposed EMW absorption mechanism of CoFe/CoFe_2_O_4_/C composites.

The electromagnetic wave absorption mechanism of this material is systematically described below (as shown in Scheme 2):

a. Impedance matching

The impedance matching is governed by the quarter-wavelength condition and the hollow architecture. The hollow microspheres adjust the phase propagation path of incident waves, enabling destructive interference between reflected and incident waves, which reduces surface reflection. Meanwhile, the geometric features of the hollow structure optimize the effective permittivity and permeability, bringing the material’s wave impedance close to that of free space. Additionally, the multiple interfaces within the hollow units modulate the local electric field distribution, balancing the contributions of dielectric and magnetic losses, thereby facilitating efficient EMW transmission across a broad frequency range.

b. Magnetic loss

Magnetic loss is considered to involve natural resonance, which may be enhanced by the surface anisotropic field and interfacial coupling. The introduction of the non-magnetic biochar phase suppresses eddy current effects by modifying the magnetic structure distribution, allowing deeper EMW penetration. The nanoscale dimensions of the magnetic particles and the heterogeneous interface design synergistically regulate magnetic anisotropy, significantly intensifying the resonance response. Furthermore, the interfacial exchange coupling between FeCo and CoFe_2_O_4_, together with multiple relaxation processes, may contribute to the magnetic energy conversion.

c. Dielectric loss

The three-dimensional conductive network of the biochar matrix provides pathways for carrier migration, contributing to conduction loss. Abundant heterogeneous interfaces, such as CoFe_2_O_4_/C, C/air, and CoFe/CoFe_2_O_4_, induce strong interfacial polarization. Meanwhile, structural defects and oxygen-containing functional groups within the carbon skeleton act as polarization centers, converting EMW energy into heat through dipolar relaxation under alternating electromagnetic fields.

d. Multiple scattering

The porous structure of biochar, in combination with the hollow CoFe/CoFe_2_O_4_ microspheres, forms a synergistic scattering system that promotes multiple internal reflections and scattering of incident waves. This extended propagation path increases the probability of energy attenuation, thereby enhancing overall absorption efficiency.

In summary, the CoFe/CoFe_2_O_4_/C composite achieves thin-layer broadband absorption through the synergy of impedance matching optimization, multiple loss mechanisms, and structure-enhanced scattering. Among the samples, CoFe/CoFe_2_O_4_/C-800 exhibits the best balance between dielectric and magnetic properties, delivering the optimal reflection loss and effective absorption bandwidth, with comprehensive performance surpassing that of most reported magnetic carbon-based composites.

## 4. Conclusions

CoFe/CoFe_2_O_4_/C composites were successfully fabricated via a simple solvothermal method combined with subsequent heat treatment. The CoFe/CoFe_2_O_4_/C-700 and CoFe/CoFe_2_O_4_/C-1000 samples exhibit poor absorption performance due to insufficient impedance matching. In contrast, the CoFe/CoFe_2_O_4_/C-800 sample achieves ideal impedance matching, synergistically integrating dielectric and magnetic losses with multiple reflection effects, thereby delivering outstanding EMW absorption properties. Specifically, at a matching thickness of 2.7 mm, a calculated *RL*_min_ of −71.4 dB is obtained, with an effective absorption bandwidth (EAB) of 3.52 GHz. When the thickness is reduced to 1.7 mm, the EAB is broadened to 5.68 GHz, covering most of the Ku band. These results demonstrate that the CoFe/CoFe_2_O_4_/C composite holds great promise as a high-performance biomass-derived EMW absorber, offering valuable insights for the design of renewable carbon-based electromagnetic attenuation materials.

## Figures and Tables

**Table 1:** Comparison of *RL*_min_, thickness, bandwidth, synthesis method, surface morphology, and filler loading for reported microwave-absorbing materials.

Sample	RL_min_(dB)	Thickness (mm)	EAB (GHz)	Synthesis Method	Surface Morphology	Filler Loading	Refs.
FeCo@CNT	−59.21	1.6	4.92	MOF-derived pyrolysis	Sea cucumber-like interconnected CNT networks	50 wt%	[54]
Fe_3_O_4_@C	−48.81	3.5	4.73	Hydrothermal calcination	Porous biochar skeleton loaded with nanoparticles	15 wt%	[36]
Rice huck@Fe	−21.8	1.4	5.6	Carbonization impregnation/reduction	Hierarchically porous carbon framework loaded with particles	25 wt%	[55]
Loofah/Fe_3_O_4_	−43.8	3	5.3	Pyrolysis	Lotus-root-like porous structure with nanoparticles	50 wt%	[7]
Coconut shell@Co	−40	5	-	Sol–gel synthesis	Porous carbon framework with nanoparticles	-	[56]
Fe/Fe_3_O_4_/C	−40.1	5.5	3.26	Pyrolysis impregnation/reduction	Hollow fiber with nanoparticles	30 wt%	[57]
LaFeO_3_	−25.6	3.5	2.2	CVD growth	Particles	50 wt%	[58]
CoFe/BPC	−53.6	2.2	4.2	In situ reduction–pyrolysis	Porous carbon matrix with nanoparticles	-	[14]
Fe/Fe_3_O_4_/BC	−30.41	2.06	2.45	Carbonization reduction	Agaric-derived porous biochar with nanoparticles	50 wt%	[59]
NiCo_2_	−55.62	3.57	4.24	Impregnation/reduction	Rice-husk-derived porous carbon with nanoparticles	30 wt%	[15]
CoFe@CoFe_2_O_4_/C	−71.4	2.7	5.68	Solvothermal–prolysis–oxidation	Lotus-leaf-derived porous carbon with hollow composite nanoparticles	50 wt%	This work

## Data Availability

The data supporting the findings of this study are available from the corresponding author on request.

## Supplementary Materials

The following supporting information can be downloaded at: https://www.mdpi.com/article/10.3390/nano16201323/s1. Figure S1: XPS spectrum of CoFe/CoFe_2_O_4_/C-800: (a) C 1s, (b) Fe 2p, (c) Co 2p, (d) O 1s, respectively; Figure S2: TEM images of CoFe/C-800 after oxidation for 15 (a,b), 30 (c,d), 60 (e,f), and 90 mins (g,h); Figure S3: (a) pore-size distribution of CoFe/CoFe_2_O_4_/C-800sample and (b) N_2_ adsorption-desorption isotherms; Figure S4: The RL curve, quarter wavelength and impedance matching curve of composite materials (a) CoFe/CoFe_2_O_4_/C-700, (b) CoFe/CoFe_2_O_4_/C-800 and (c) CoFe/CoFe_2_O_4_/C-1000.
