# Supplementary material for: Boosting Microwave Absorption via Hollow CoFe/CoFe2O4 Nanoparticles Anchored on Lotus-Leaf-Derived Biochar
Source: Nanomaterials (Basel). 2026 Oct 20;16(20):1323. doi: 10.3390/nano16201323 (PMC13707890; doi:10.3390/nano16201323)
Supplement: Supplementary file 1 [file nanomaterials-16-01323-s001.zip › nanomaterials-4586022-supplementary.pdf]

Supporting materials

Boosting Microwave Absorption via Hollow $CoFe/CoFe_2O_4$ Nanoparticles Anchored on Lotus Leaf-Derived Biochar

Feng Han [a], Maomao He [a], Hongchun Yu [a], Luran Zhang [a, *]

[a] Yunnan Key Laboratory of Electromagnetic Materials and Devices, National Center for International Research on Photoelectric and Energy Materials, School of Materials and Energy, Yunnan University, Kunming 650500, China

* Contact information: Luran Zhang, zhangluran@ynu.edu.cn

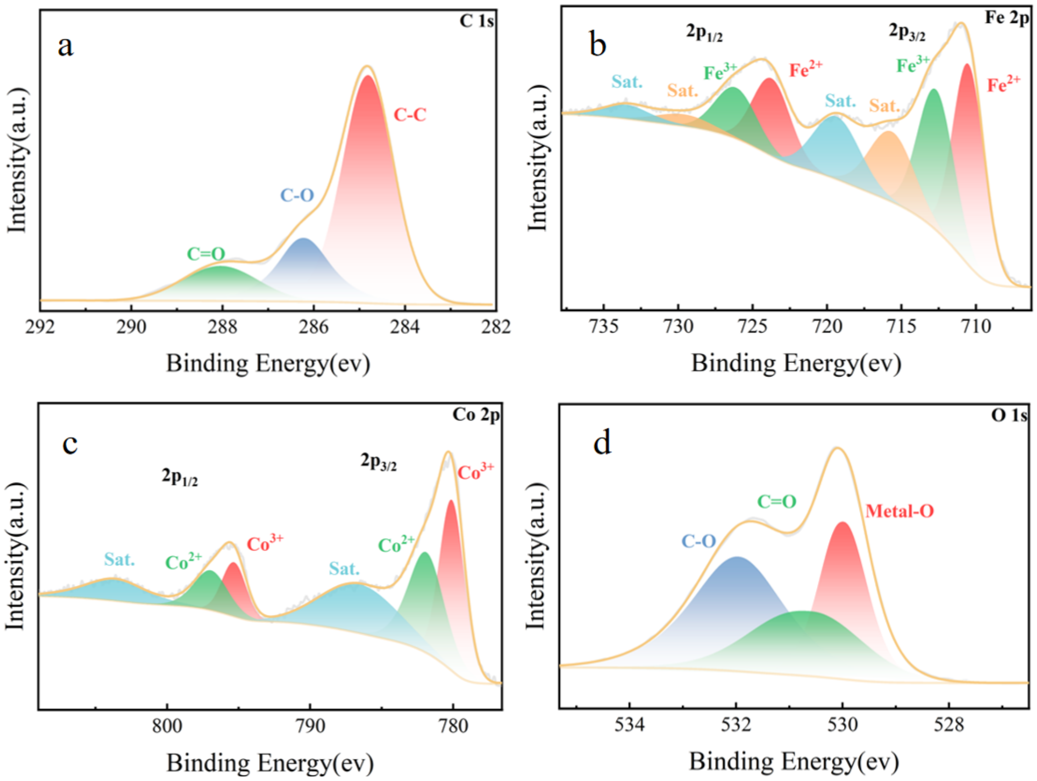

Fig. S1. XPS spectrum of $CoFe/CoFe_2O_4$/C-800: (a) C 1s, (b) Fe 2p, (c) Co 2p, (d) O 1s, respectively

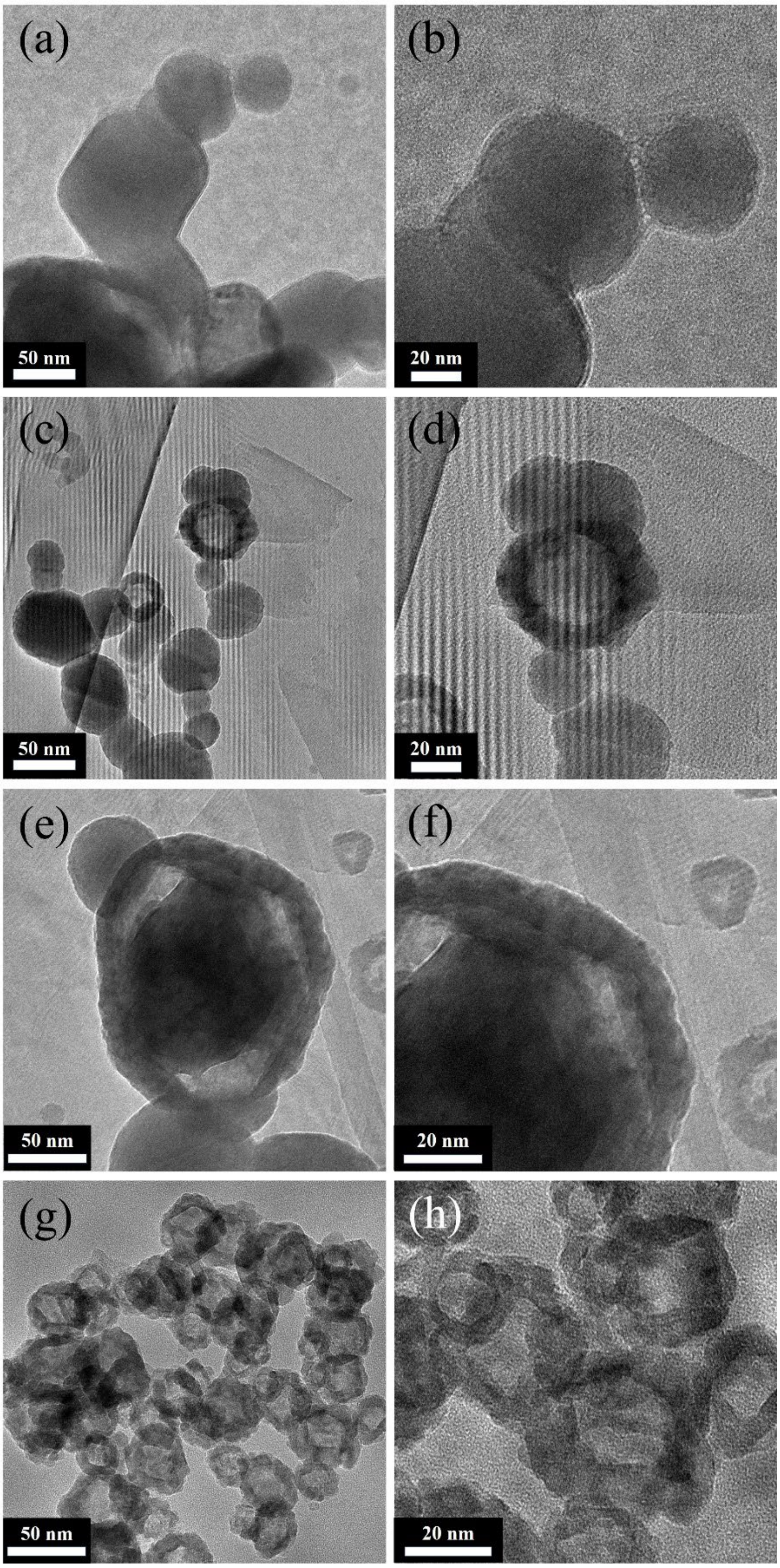

Fig. S2. TEM images of CoFe/C-800 after oxidation for 15(a, b), 30(c, d), 60(e, f), and 90 mins(g, h).

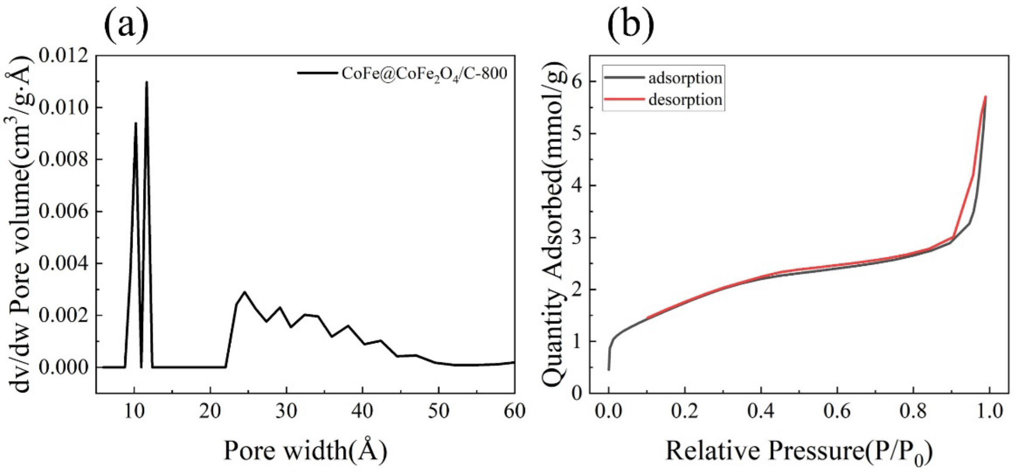

Fig. S3 (a) pore-size distribution of CoFe/CoFe2O4/C-800sample and (b) N_2 adsorption-desorption isotherms.

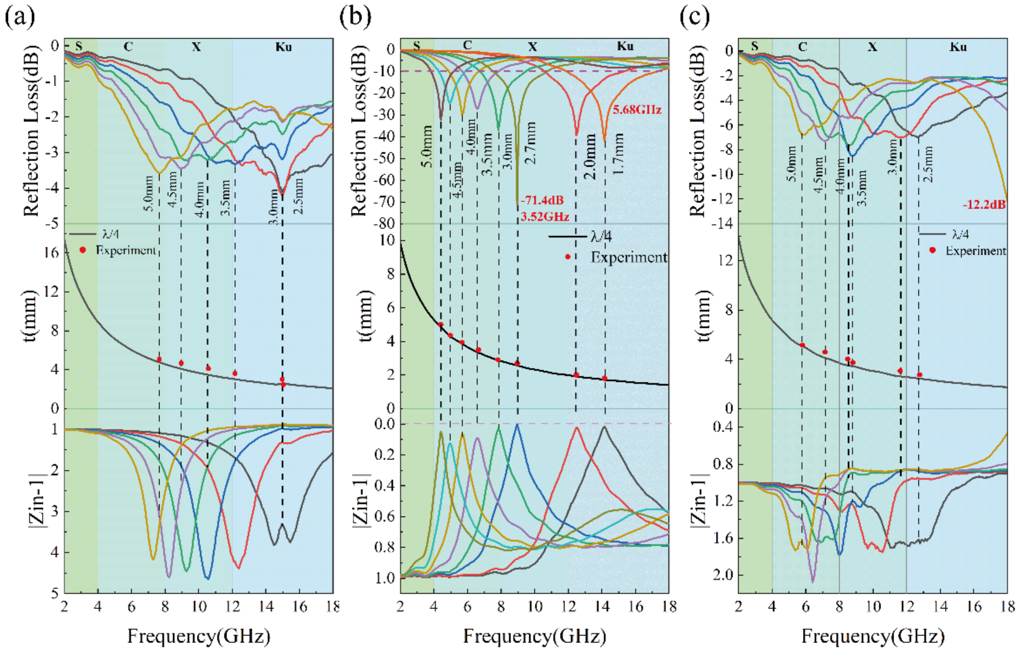

Fig. S4. The RL curve, quarter wavelength and impedance matching curve of composite materials (a) CoFe/CoFe2O4/C-700, (b) CoFe/CoFe2O4/C-800 and (c) CoFe/CoFe2O4/C-1000.
